# Microbial approaches for the assessment of toothpaste efficacy against oral species: A method comparison

**DOI:** 10.1002/mbo3.1271

**Published:** 2022-03-16

**Authors:** Pune N. Paqué, Lamprini Karygianni, Julien Kneubuehler, Lorenzo Fiscalini, Daniel B. Wiedemeier, Marcel Müller, Thomas Attin, Thomas Thurnheer

**Affiliations:** ^1^ Clinic for Conservative and Preventive Dentistry, Center of Dental Medicine University of Zurich Zurich Switzerland; ^2^ Privat Dental Praxis Zurich Switzerland; ^3^ Statistical Services, Center of Dental Medicine University of Zurich Zurich Switzerland

**Keywords:** agar plug assay, antimicrobial effect, CFU, dentifrices, disc diffusion assay, fluoride

## Abstract

Antibacterial properties of toothpastes enable chemical plaque control in limited‐access tooth regions that are mechanically not sufficiently reached by toothbrushes. Therefore, this study aimed to compare different microbial methods to assess antimicrobial toothpaste properties and evaluate different toothpastes in terms of their antibacterial efficacy against different oral microorganisms in an in vitro setting. Six toothpaste suspensions with varying antibacterial supplements were applied to a multispecies biofilm model (*Actinomyces oris, Candida albicans, Fusobacterium nucleatum, Streptococcus oralis*, and *Streptococcus mutans*) as well as to each microorganism. A culture method was used to assess the anti‐biofilm effects and two different agar diffusion assays were performed for testing the antimicrobial effect on each microorganism. The measurements of the culture and diffusion analyses were statistically normalized and compared and toothpastes were ranked according to their antimicrobial efficacy. The results of both agar diffusion assays showed a high correlation across all tested species (Spearman correlation coefficients *ρ*_s_ > 0.95). The results of the multispecies biofilm model, however, substantially differed in its assessment of antibacterial properties (*ρ*_s_ ranging from 0.22 to 0.87) compared to the results of both diffusion assays. Toothpastes with amine fluoride (with and without stannous fluoride), and toothpastes with triclosan resulted in the highest antimicrobial efficacy. Activated carbon supplements in toothpastes were comparable in their antimicrobial action to the negative control NaCl. The appropriate selection of a broad range of oral microorganisms seems crucial when testing the chemical impact of toothpaste and toothpaste supplements.

## Graphical abstract

The appropriate selection of a broad range of oral microorganisms seems crucial when testing the chemical impact of toothpaste and toothpaste supplements. This study aimed first to compare diffusion assays and a multispecies biofilm experiment to assess antimicrobial toothpaste properties and later evaluate different toothpastes on their antibacterial efficacy against different oral microorganisms in vitro.

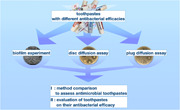

## 1 INTRODUCTION

The improvement of self‐performed plaque control is gaining increasing attention to maintain oral health and prevent diseases, such as caries and periodontitis (Kilian et al., 2016). Oral diseases are caused by biofilms, which adhere to the tooth and epithelial surfaces in the oral cavity (Marsh & Bradshaw, 1995). Oral biofilms comprise diverse oral microorganisms and a self‐produced cell‐surrounding matrix of hydrated extracellular polymeric substances (EPS) (Flemming & Wingender, 2010). Sufficient self‐care plaque control is the most important measure to avoid shifts in the oral microbiome toward more pathogenic microorganisms, thereby preventing complex dysbiotic diseases (Hajishengallis et al., 2012). For this purpose, toothbrushing twice a day with fluoridated toothpaste is considered the “gold standard” recommendation of oral hygiene. This oral hygiene regimen comprises a chemo‐mechanical plaque control, applying an electric or manual toothbrush to clean the teeth and toothpaste to reach the remaining surfaces that are not directly affected by the bristles (Chapple et al., 2015). The best evidence to reduce plaque scores, bleeding scores, and probe pocket depth in these limited‐access areas has been described by mechanical cleaning with interdental brushes compared with other interdental cleaning devices as adjuncts to plain toothbrushing (Slot et al., 2008, 2020). Interestingly, toothpastes that facilitate chemical supplementation to mechanical cleaning are most commonly studied in terms of their mechanical properties after brushing, such as abrasive effects, surface roughness, and cleaning efficacy on dental hard tissues (Addy & Hunter, 2003; Tawakoli et al., 2015). Compared with these mechanical properties, only little data is available for chemical adjuncts to complement plaque control (Chapple et al., 2015). Nonetheless, chemical supplements in toothpaste could not only benefit caries‐active patients but also patients with impaired accessible tooth regions, due to orthodontic treatment or anatomic conditions (Pithon et al., 2015): Patients with special needs and impaired manual dexterity could also benefit from plaque‐ and gingivitis‐reducing agents applied as a daily preventive measure (Christensen et al., 2005). These chemical agents could be implemented in toothpaste and/or mouth rinses, although direct comparisons regarding the delivery format of the chemical agents are lacking to date (Polak et al., 2015). The frequent use of toothpaste exceeds the use of mouth rinses and would therefore provide a suitable vehicle for antimicrobial agents during self‐performed plaque control (Polak et al., 2015). The most important substances employed in toothpastes remain fluorides for caries control, although toothpastes also function as delivery vehicles for antimicrobial agents and thereby plaque‐ and gingivitis‐reducing agents. In this context, significant differences have been detected between different toothpastes (Arweiler et al., 2018; de Oliveira Carvalho et al., 2020; Wade et al., 1997). The antimicrobials in toothpastes have been shown to remain bio‐available in plaque‐left‐behinds after brushing (Otten et al., 2011), a fact that highlights the importance of a suitable toothpaste composition during self‐performed plaque control.

Nevertheless, the broad market supply of toothpastes, as well as the rapidly changing diversity of toothpastes, complicates evidence‐based recommendations for patients. The introduction of standardized laboratory tests would simplify the evaluation of different antibacterial adjuncts to toothpastes. Although there are many different microbial methods to evaluate the antimicrobial potential of toothpastes, their effectiveness and comparability have yet not been systematically analyzed.

Therefore, we aimed to apply three different frequently used experimental setups to analyze the antimicrobial efficacy of toothpastes with different microbial agents. The primary aim was to compare two different microbial approaches, namely culture analysis of multi‐species oral biofilms with two different agar diffusion assay methods. The secondary aim was the overall comparison of different toothpastes and their antimicrobial efficacy based on all three assays. The null hypothesis assumed that all toothpastes exhibit the same effects on all species, irrespective of the microbial analysis applied.

## 2 MATERIALS AND METHODS

### 2.1 Biofilm experiments

#### 2.1.1 Biofilm formation

Five‐species biofilms containing *Actinomyces oris* OMZ 745, *Candida albicans* OMZ 110, *Fusobacterium nucleatum* KP‐F2 (OMZ 596), *Streptococcus oralis* SK 248 (OMZ 607), and *Streptococcus mutans* UA159 (OMZ 918) were used to evaluate antimicrobial effects of different toothpastes. Biofilm formation was based on previous studies as already described in detail (Karygianni et al., 2020; Shapiro et al., 2002; Thurnheer et al., 2003). In brief, each strain was inoculated in a modified fluid universal medium (mFUM) (Guggenheim et al., 2001) and incubated overnight at 37°C anaerobically. After 16 h, new precultures were produced by pipetting 0.8–1.5 ml of the overnight microbial suspension in fresh mFUM for 5–7 h. Each suspension was adjusted to the optical density OD_550_ = 1.0 (Spectra Max, Molecular Devices) and then mixed in equal volumes to produce the inoculum. Hydroxyapatite discs (*n* = 24, HA; Ø 9 mm; Clarkson Chromatography Products Inc.) were preconditioned with whole unstimulated saliva for 4 h to induce pellicle formation according to Guggenheim et al. (2001). For this purpose, the whole unstimulated saliva of several volunteers was collected, pooled, and centrifuged to produce processed saliva. The detailed protocol was described previously (Guggenheim et al., 2001). The HA discs were then covered with 1.6 ml growth medium (1120 µl of processed saliva and 480 µl mFUM supplemented with Sørensen's buffer at a final of pH 7.2 + 0.3% glucose) and 200 µl of the inoculum to induce biofilm formation. The discs were then incubated anaerobically for 64 h. Based on earlier work (Guggenheim et al., 2001), the carbohydrate concentration of mFUM was changed with the medium change after 16 h to 0.15% glucose and 0.15% sucrose.

#### 2.1.2 Treatment with toothpastes

Six different toothpastes (Table 1) were used to assess the antimicrobial effects on the aforementioned oral species. Toothpaste suspensions were produced by mixing toothpastes with 0.9% saline solution (NaCl) in a ratio of 1:2 by vortexing. After 16, 20, 24, 40, 44, and 48 h of biofilm formation, biofilms were exposed to 1 ml of each toothpaste suspension in triplets. A 0.2% chlorhexidine solution (CHX; Chlorhexamed, GlaxoSmithKline Consumer Healthcare GmbH & Co. KG) served as a positive control, while a saline solution (NaCl) served as a negative control to mimic untreated biofilms. Biofilms were treated each time for 1 min with the respective suspensions or controls and then dipped three times in NaCl to remove the remaining suspension and nonadherent microorganisms. After treatment, the biofilms were transferred to either fresh medium (after 16 and 40 h) or the earlier‐used medium (all other time points), to foster a stable and resistant biofilm (Balouiri et al., 2016). Biofilms were harvested after 64 h of biofilm formation and culture analyses were conducted.

**Table 1:** Overview of all six tested toothpastes and their antimicrobial agents and terminology throughout the article

Toothpaste/rinses	Antibacterial substances [*terminology*]	Company
Colgate Total® Original	Triclosan [*triclosan‐TP*]	GABA Switzerland^a^, Colgate‐Palmolive Company
Curaprox Black is White	Activated carbon [*activated carbon‐TP*]	Curaden International AG^b^
Elmex® Kariesschutz	Amine fluoride [*AmF‐TP*]	GABA Switzerland^a^, Colgate‐Palmolive Company
Meridol®	Amine fluoride/stannous fluoride [*AmF/SnF2‐TP*]	GABA Switzerland^a^, Colgate‐Palmolive Company
Parodontax Original	Essential oils [*essential oil‐TP*]	GlaxoSmithKline^c^
Signal Antikaries	Sodium fluoride *[NaF‐TP]*	Unilever^d^

^a^ Therwil, Switzerland.

^b^ Kriens, Switzerland.

^c^ Rotkreuz, Switzerland.

^d^ Thayngen, Switzerland.

#### 2.1.3 Culture analyses

**Figure A3:**
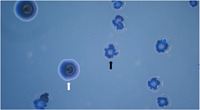
The image shows a mixed culture on Mitis Salivarius agar. With some experience, *Streptococcus mutans* (white arrow) and *Streptococcus oralis* (black arrow) can be distinguished from each other, as the former forms smooth colonies on this agar, while *S. oralis* shows a so‐called blackberry morphology. (Bertolini et al., 2015; Johnson et al., 1974; Lemos et al., 2013)

After 64 h of biofilm formation, the discs were dipped three times in NaCl to remove nonadherent microorganisms from the surface. The adherent biofilms on the discs were then suspended in 1 ml of 0.9% NaCl by vortexing for 1 min and further sonification for 5 s (Branson B‐12, Branson). Serial dilutions (10^0^–10^4^) were prepared from the bacterial suspensions using 0.9% NaCl, of which 50 µl aliquots were plated out on different agar plates using a spiral diluter (Eddy Jet 2 Diluter; IG Instruments). Columbia blood agar plates (CBA; Oxoid Ltd.), enriched with 5% whole human blood (Blood Donation Swiss Red Cross), were used to determine total colony‐forming unit (CFU) counts. Selective agar plates were used to determine the CFU counts of each biofilm species, as described elsewhere (Guggenheim et al., 2001; Klinke et al., 2011). Besides the total CFU counts, the CBA plates were also used to determine *A. oris*. *S. mutans* and *S. oralis* were both quantified on Mitis Salivarius Agar (Difco Laboratories, Inc.) supplemented with 0.001% (w/v) sodium tellurite). *C. albicans* was quantified on BIGGY Agar plates (BBL; Becton Dickinson). The CBA plates were anaerobically incubated at 37°C, while MITIS and BIGGY plates were aerobically incubated at 37°C. After 72 h of incubation, CFU counts were determined on the plates, differentiating the species by colony morphology (Figure A3).

### 2.2 Diffusion assays

#### 2.2.1 Agar disc diffusion assay

The mFUM agar plates were produced by mixing the medium with 1% agar (Agar Nobile, Beckton Dickinson). The resulting mFUM agar solution was autoclaved (121°C, 15 min) and cooled to 50°C before filling the plates (SPL life sciences, Crystal‐grade Polystyrene Gamma sterilized, 150 ×20 mm; Semadeni AG) with 40 ml of the agar solution. A selective agar was used for *F. nucleatum* analog to the mFUM agar, mixing 13.7 g Fastidious Anaerobe Agar (BAG; Neogen Corporation UK) with 300 ml Aqua dest.

Before experimentation, precultures of each bacterial species (*A. oris*, *C. albicans*, *F. nucleatum*, *S. oralis*, and *S. mutans*) were inoculated as described above to reach comparable optical densities of OD_550_ = 1.0. Each microbial suspension was dispensed on the freshly produced agar plates. Five sterile filter paper discs (Ø 9 mm; Gel‐Blotting Paper, Whatman™, Fisher Scientific Sa) were applied on each plate and immediately covered with 100 µl of each test solution. Toothpaste suspensions (1:2 in NaCl) were tested in triplets per species and triplets per plate (*n* = 9). Each selective plate comprised a three times test solution, the positive control, and the negative control. Incubation followed at 37°C for 24 h (*C. albicans*) and 48 h (*A. oris*, *F. nucleatum*, *S. oralis*, and *S. mutans*) anaerobically in jars with gas‐paks (GENbox anaer, bioMérieux® Sa). After incubation, zones of inhibition around the discs were measured on the tightest diameter using a digital caliper (Holex Electronic AG).

#### 2.2.2 Agar plug diffusion assay

The agar plug diffusion assay was applied analog to the agar disc diffusion assay. Freshly produced plates (mFUM agar and Fastidious Anaerobe agar) were covered with each of the bacterial suspensions (*A. oris*, *C. albicans*, *F. nucleatum*, *S. oralis*, and *S. mutans* at OD_550_ = 1.0). Five plugs were cut into each plate using a sterile punch (Ø 9 mm) (Balouiri et al., 2016). Before incubation at 37°C for 24 h, 100 µl of each toothpaste suspension (triplets per plate), the positive and negative controls were pipetted into the plugs. After 24 h (*C. albicans*) and 48 h (*A. oris*, *F. nucleatum*, *S. oralis*, and *S. mutans*) of cultivation under anaerobic conditions, the zones of inhibition around the plugs were measured as described above.

### 2.3 Statistical analysis

The biofilm data were analyzed using Kruskal–Wallis rank‐sum tests followed by pairwise comparisons between toothpastes according to Conover. The resulting *p* values for the global tests and the posthoc tests were adjusted for the false discovery rate of 5% using the Benjamini and Yekutieli correction. A resampling approach was chosen to take into account the potential lack of independence between the observations due to the experimental setup. The test results on the original data set are only reported as significant if the adjusted *p* value is less than the chosen significance level (5%) and the resampling rate is greater than 95% (proportion of significant test results over 1000 resamples). For the comparison of methods, the data of the culture analyses and the agar diffusion methods were scaled to a consistent range; that is, biofilm data were first log10(*x* + 1) transformed, and then all data were normalized and transformed to numbers between 0 and 1, with 0 being the lowest antibacterial efficacy and 1 the highest per method across all species. The coefficient of variation was calculated for each species and toothpaste overall assays. The methods were further compared by applying a standardized ranking of the antimicrobial efficacy (0 = no antimicrobial effect, 1 = maximum antimicrobial effect) of distinct toothpastes for each species and total CFU (multiple observations for the same toothpaste were aggregated by the median). The Spearman correlation coefficient *ρ*_s_ of the standardized ranking between different assays was then calculated for each species and total CFU. Furthermore, multidimensional scaling (based on Euclidean distances) on the standardized ranking of the three assays was applied separately for each species and presented as a two‐dimensional chart. All calculations were performed with the statistical software R (Core Team, 2020) using the following packages (Mangiafico, 2020; Millard, 2013; Pohlert, 2014; Wickham, 2016; Wickham & Seidel, 2020). The raw data of all experiments are provided in supplemental material at https://doi.org/10.6084/m9.figshare.19160843.

## 3 RESULTS

### 3.1 Antimicrobial efficacy of toothpastes against biofilms

**Figure 1:**
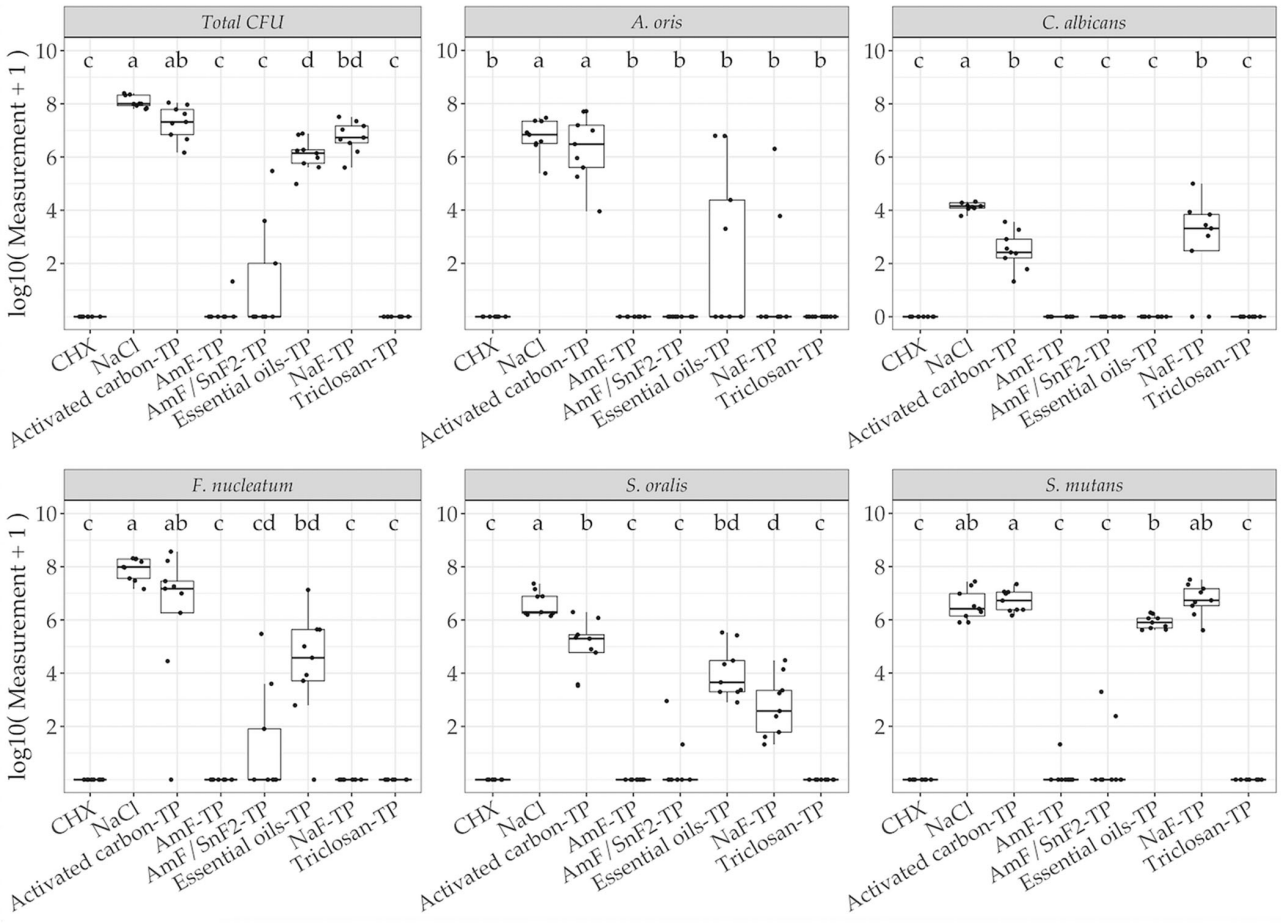
Boxplots with the median values and interquartile ranges of the biofilm data log10(*x* + 1) present the antimicrobial efficacy of all six tested toothpaste suspensions for the total colony‐forming unit and species‐specific results (*n* = 9). Means with the same letter are not significantly different from each other (*p* < 0.05)

Species‐specific differences were observed in the antimicrobial efficacy of the six tested toothpastes against in vitro multispecies biofilms. The total CFU counts resulted in high antimicrobial efficacy for the amine fluoride toothpaste (AmF‐TP), the triclosan‐TP, and the positive control (0.2% CHX). The activated carbon‐TP did not significantly differ from the negative control (0.9% NaCl). Similarly, low antimicrobial efficacies were shown for the essential oil‐TP and sodium fluoride toothpaste (NaF‐TP). Comparing the total CFU counts to a species‐specific level, all toothpastes exhibited similar antimicrobial efficacy on both *S. mutans* and *S. oralis*, while the essential oil‐TP and NaF‐TP yielded different antimicrobial efficacy on *A. oris*, *C. albicans*, and *F. nucleatum*. The activated carbon‐TP followed by the essential oil‐TP and NaF‐TP exhibited the lowest antimicrobial efficacy overall species, comparable to the negative control (untreated biofilms). The highest antimicrobial efficacy was shown for the AmF‐TP and triclosan‐TP, followed by the AmF/SnF_2_‐TP (Figure 1). The results of the diffusion assays are separately shown as boxplots in Appendix A.

### 3.2 Comparison of microbial assays for antimicrobial efficacy of toothpastes

**Figure 2:**
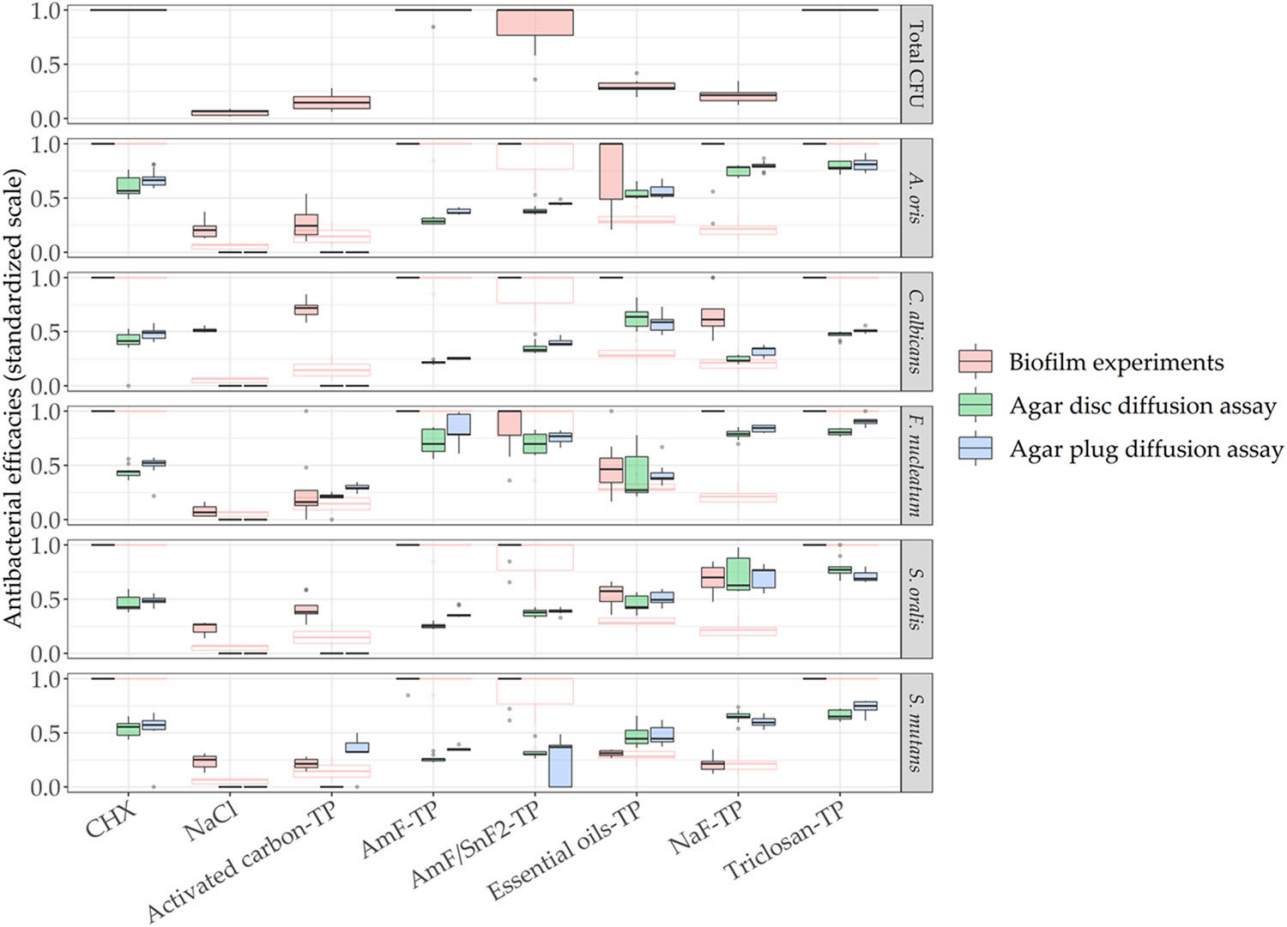
Boxplots with the median values and interquartile ranges present the antimicrobial efficacy of all six tested toothpaste suspensions with different microbial assays (*n* = 9). Total colony‐forming unit (CFU) counts over all species are available for the biofilm experiments (red) and added as a reference on the species‐wise outcomes. Data for the agar disc diffusion assay are shown in green and for the agar plug diffusion assay in blue

To enable interassay comparisons, all data were transformed and standardized to values between 0 and 1 (0 = no antimicrobial efficacy, 1 = strong antimicrobial efficacy). The standardized outcomes of all assays were compared graphically (Figure 2). The total CFU data show strong antimicrobial efficacy for 0.2% CHX, triclosan‐TP, AmF‐TP, and slightly less pronounced efficacy for AmF/SnF_2_‐TP. Species‐specific results for the biofilm experiment reflected the following pattern: *A. oris*, *C. albicans*, *S. oralis*, and *S. mutans* showed similar antimicrobial effects after treatment with 0.2% CHX, triclosan‐TP, AmF‐TP, and AmF/SnF_2_, while *F. nucleatum* showed inconsistent outcomes after AmF/SnF_2_‐TP exposure, resulting in high interquartile ranges. Moderate antimicrobial efficacy was shown for the essential oil‐TP and NaF‐TP. However, analysis of species‐specific CFU data for *A. oris* and *C. albicans* revealed higher antimicrobial efficacy compared with that against *S. oralis* and *S. mutans*. The essential oil‐TP showed a moderate antimicrobial efficacy against *F. nucleatum*, while NaF‐TP demonstrated high efficacy against *F. nucleatum*. The negative control (0.9% NaCl) and the activated carbon‐TP exhibited no antimicrobial efficacy in the biofilm experiments for most species and the total CFU counts. Activated carbon‐TP yielded a moderate antimicrobial efficacy against *C. albicans*.

The diffusion assays revealed comparable antimicrobial actions, showing high efficacy for CHX, triclosan‐TP, while less pronounced antimicrobial efficacy was measured for the essential oil‐TP and NaF‐TP. Different trends were observed for AmF‐TP and AmF/SnF_2_‐TP: both toothpastes exhibited strong antibacterial actions against *F. nucleatum*, but not equally strong against other species (Figure 2). Table 2 depicts the correlation coefficients between the methods and presents the similarity of the microbial methods for the quantification of each species. The correlation coefficients for different species among the diffusion methods resulted in a minimum of 0.95 (*S. oralis*), highlighting their overall close relation regarding outcomes.

**Table 2:** Species‐wise correlation coefficient *ρ*_s_ of the applied microbial assays

	*Actinomyces oris*	*Candida albicans*	*Fusobacterium nucleatum*	*Streptococcus oralis*	*Streptococcus mutans*
Biofilm/disc assay	0.75	0.71	0.87	0.56	0.23
Biofilm/plug assay	0.75	0.71	0.87	0.42	0.22
Disc/plug assay	0.98	1	1	0.95	0.99

*Note*: Disc assay, agar disc diffusion assay; Plug assay, agar plug diffusion assay.

**Figure 3:**
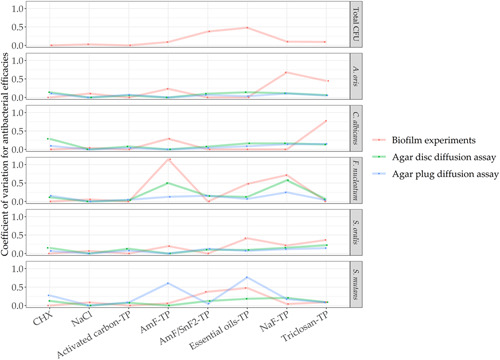
Coefficient of variation is shown for antimicrobial efficacy in the biofilm experiments (red), agar disc diffusion assay (green), and agar plug diffusion assay (blue)

The precision of the assays is presented graphically in Figure 3. The coefficient of variation highlights differences between the assays for each toothpaste and species. High coefficients of variation were observed for most assays in AmF‐TP, NaF‐TP, and essential oil‐TP. In particular, the counts of *A. oris* (NaF‐TP, triclosan‐TP), *C. albicans* (AmF‐TP, triclosan‐TP), *S. oralis* (essential oil‐TP), and *F. nucleatum* (AmF‐TP, essential oil‐TP, NaF‐TP) depicted strong variations in the biofilm experiments. Similarly, strong variations of coefficients were found in the diffusion assays for *F. nucleatum* (agar disc diffusion assay) and *S. mutans* (agar plug diffusion assay) after exposure to AmF‐TP and essential oil‐TP.

### 3.3 Ranking of standardized antimicrobial efficacy of microbial assays

**Figure 4:**
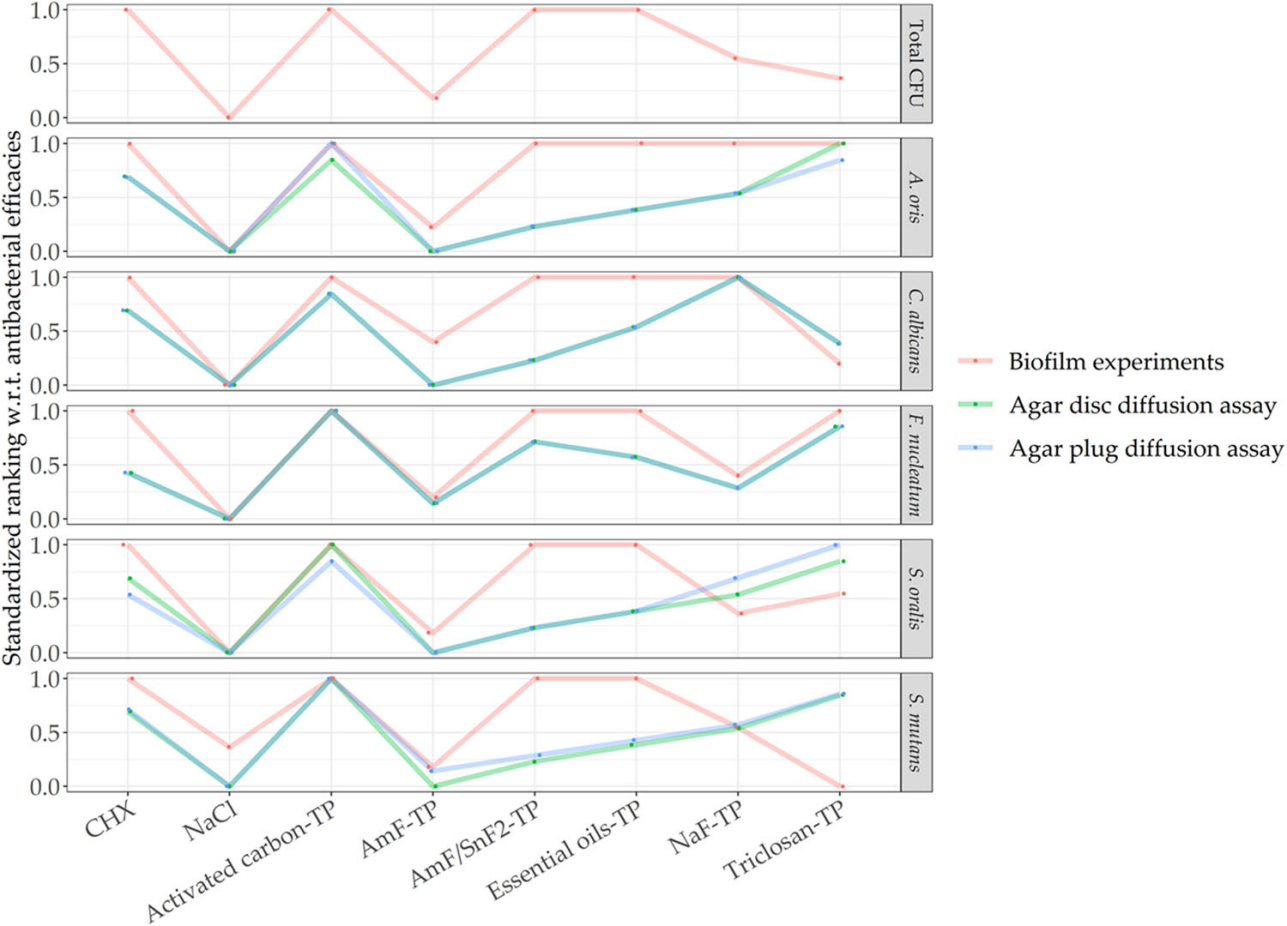
Comparison of standardized ranking of the antimicrobial efficacy of distinct toothpastes across different microbial assays and species. Data obtained by the biofilm experiments are shown in red, by agar disc diffusion assay in green, and agar plug diffusion assay in blue

The standardized ranking of antimicrobial efficacy of the applied assays (Figure 4) mainly showed differences between the biofilm experiments and the diffusion assays regarding toothpastes with stannous fluoride (AmF/SnF_2_‐TP) and essential oils (essential oil‐TP). Comparing the antimicrobial actions as revealed by the biofilm experiments to the antimicrobial efficacy estimated by both diffusion assays resulted in the following differences detected regarding toothpastes and species: AmF/SnF_2_‐TP, essential oil‐TP, and NaF‐TP differed in their antimicrobial efficacy against *A. oris* (correlation coefficient *ρ*_s_ of 0.75); AmF‐TP, AmF/SnF_2_‐TP, and essential oil‐TP against *C. candida* (*ρ*_s_ of O.71) and *S. oralis* (*ρ*_s_ of 0.56 biofilm/disc and *ρ*_s_ of 0.41 biofilm/plug); AmF/SnF_2_‐TP and the essential oil‐TP against *F. nucleatum* (*ρ*_s_ of 0.87); and AmF/SnF_2_‐TP, essential oil‐TP, and triclosan‐TP against *S. mutans* (*ρ*_s_ of 0.23 biofilm/disc and *ρ*_s_ of 0.22 biofilm/plug). The weakest correlation between the methods was therefore measured between the assays regarding *S. mutans* (Table 2).

### 3.4 Similarity of toothpaste ranking over all assays

**Figure 5:**
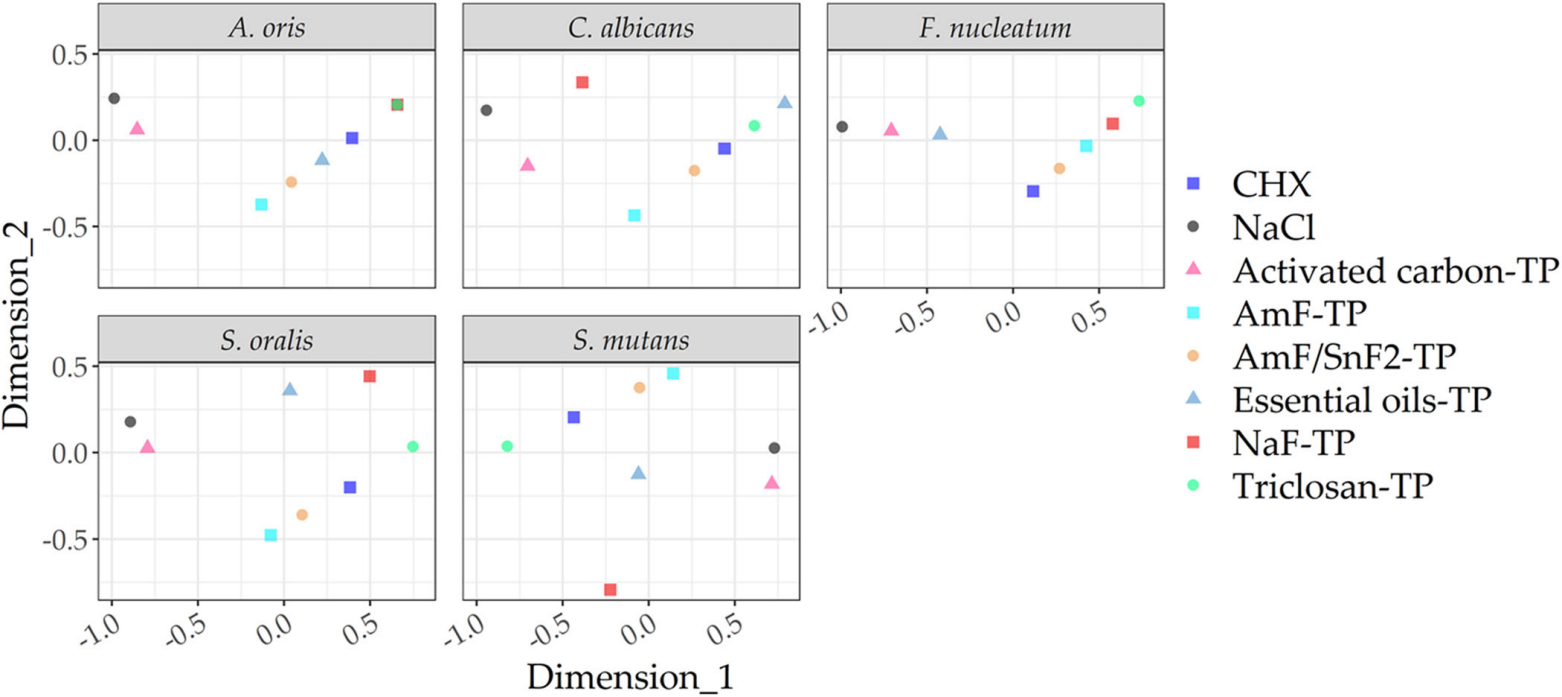
Comparison of toothpastes aggregated over different assays by multidimensional scaling. The overall similarity in antibacterial efficacy of each toothpaste across all three methods is illustrated in a two‐dimensional chart. The figure axes were rescaled in a way that the spatial proximity of the toothpastes shows the overall similarity of their antimicrobial action against the respective species

Figure 5 visualizes the similarity of the efficacy ranking and thus the antibacterial efficacy of each individual toothpaste in a two‐dimensional chart. The closer that the toothpastes are located, the stronger the similarity of their antimicrobial action against the respective species as measured by all three methods together. The AmF‐TP and AmF/SF_2_‐TP appeared most often close to each other. The negative control (0.9% NaCl) and the activated carbon‐TP also appeared close to each other, indicating similar antimicrobial efficacy in most of the tested species (Figure 5).

## 4 DISCUSSION

In our study, the comparison of three different microbial methods (culture analysis, agar disc diffusion assay, agar plug diffusion assay) to evaluate the antimicrobial potential of six different toothpastes revealed similar antimicrobial trends against the tested oral microorganisms correlating with the chemical properties of the toothpastes. Interestingly, discrepancies of the toothpaste antimicrobial efficacy were found at a species‐specific level within the same microbial assays. However, the interdiffusion‐array comparison suggested a comparable toothpaste ranking of antimicrobial efficacy. Thus, the null hypothesis was rejected.

Clinical studies on the chemical effectiveness of toothpastes are rare and most notably due to frequent changes in the products and their formulations often become antiquated as soon as they are published. Thus, laboratory analyses are preferentially applied to screen diverse toothpaste properties, besides their mechanical action. Unfortunately, the lack of standardized procedures for quality control limits the interpretation and comparison of different studies in this field. Applied laboratory methods to evaluate the antimicrobial properties of toothpastes vary to a strong degree (de Oliveira Carvalho et al., 2020; Friesen et al., 2017; Vanni et al., 2015). Multispecies biofilm models mimic the interaction of biofilm‐embedded microorganisms toward antimicrobial agents in toothpaste or rinses (Otten et al., 2011; Vanni et al., 2015). Nonetheless, most toothpaste evaluation studies are based on varying diffusion assays using discs or plugs with a single‐ or multispecies approach (Karadaglioglu et al., 2019; Randall et al., 2015). Therefore, we compared the results of an in vitro multispecies biofilm model with two agar diffusion assays yielding species‐specific data (Figure A3). Collectively, the ranking of the antimicrobial toothpaste properties resulted in similar outcomes, independent of the microbial assay applied. In particular, the choice of a specific diffusion assay (agar disc or agar plug diffusion assay) seems insignificant for all species used in this study. Interestingly, the method comparison between culture analysis and both diffusion assays only revealed a very weak correlation for *S. mutans*. This seems to hold strong importance because *S. mutans* is frequently used in microbial analyses of antimicrobial agents using single‐species diffusion assays (Gutierrez et al., 2017; Lewinstein et al., 2005; Su et al., 2018). Our results suggest that statements concerning antimicrobial efficacy of products do not necessarily seem conclusive if solely based on *S. mutans* diffusion assays. Thus, the use of a broad range of oral microorganisms or a multispecies biofilm ensures a significant antimicrobial evaluation. A potential limitation of our study is the grading of the toothpaste according to the results of the biofilm model, by assuming that the biofilm mimics the clinical situation more closely than its planktonic counterparts in the diffusion assays. A clinical validation with different clinical cohorts, as well as the implementation of mechanical brushing, would serve as a valuable comparison and reveal the approach by the different in vitro models more precisely. This was, however, not the scope of our study and would lead to the disproportional effort. The same applies to other cost‐intensive methodologies, such as quantitative polymerase chain reaction (qPCR), which would produce more accurate data; however, live and dead cells are both detectable with qPCR. Therefore, this method cannot efficiently answer the primary question of the present report. Another more general limitation in the methodology is the application of different diffusion assays for the testing of toothpastes. It remains unclear whether all active toothpaste ingredients diffuse homogeneously through the discs and agar.

All three microbial assays applied resulted in low antimicrobial efficacy for the toothpaste with activated carbon and the negative control. Activated carbon is currently added to diverse toothpastes and mouthwashes, and is also incorporated in toothbrush bristles (Greenwall et al., 2019; Thamke et al., 2018). Nonetheless, evidence is lacking for its effect, particularly regarding the antimicrobial properties of activated carbon in toothpastes (Brooks et al., 2017). The mode of action of activated carbon (also termed charcoal) is based on its high absorbent qualities, which are used in medicine to treat poisoning and overdoses of medication (Juurlink, 2016).

The most extensively studied active ingredients of toothpastes are triclosan, followed by stannous fluoride (Valkenburg & Else Slot, 2020). Both supplements exhibit high antimicrobial activity, which is in line with the outcomes of our study. The strong effects of triclosan are described as an interplay between triclosan, its copolymer, and often adjuncts of zinc citrate, which together inhibit glycolysis, bacterial proteases, and the interleukin‐induced production of prostaglandin E_2_ with an additional overall reduction of Gram‐positive and Gram‐negative bacteria by damages in the integrity of the plasma membrane (Zuckerbraun et al., 1998).

Most studies comparing the additive effect of antimicrobial adjuncts use sodium fluoride toothpastes as a control (NaF) (Fernandez et al., 2017; Wade et al., 1997). However, in our experiments, moderate to high antimicrobial efficacy against most tested oral microorganisms were found. This outcome might be explained by the synergistic effects of other ingredients in the respective toothpastes. For instance, sodium lauryl sulfate is known to contribute effectively to the antimicrobial action of toothpastes (Randall et al., 2015).

Another rather less effective toothpaste supplement in our study was the essential oil toothpaste. However, other studies have revealed positive effects if essential oils were implemented in fluoride‐free toothpastes to improve inhibitory effects against caries‐associated microorganisms (de Oliveira Carvalho et al., 2020). While essential oils have mostly been investigated as mouth rinses to prevent oral diseases, their implementation in toothpastes with varying composition and concentration has not yet been sufficiently investigated (Jackson, 1997). The insufficient degree of evidence concerning many antimicrobial agents in toothpaste—despite being broadly applied and commercialized—highlights the need for standardized assays to facilitate their efficient evaluation and benefit.

## 5 CONCLUSIONS

The six tested toothpastes differed in their antimicrobial activity and the results differed depending on the method used. Most toothpastes affected oral microorganisms to a different degree, resulting in species‐specific differences for each toothpaste. Differences in the outcome of the microbial methods were observed for *S. mutans*, followed by *S. oralis*, *C. albicans*, and *A. oris*. Hence, the selection of a broad range of oral microorganisms as single‐ or multispecies biofilms is recommended for the efficient evaluation of antimicrobial properties of toothpastes.

## CONFLICTS OF INTEREST

The authors declare no conflicts of interest.

## ETHICS STATEMENT

None required.

## AUTHOR CONTRIBUTIONS

**Pune N. Paqué**: Conceptualization (equal); writing—original draft (lead); writing—review and editing (equal). **Lamprini Karygianni**: Conceptualization (equal); writing—original draft (supporting); writing—review and editing (equal). **Julien Kneubuehler**: Writing—review and editing (equal). **Lorenzo Fiscalini**: Writing—review and editing (equal). **Daniel B. Wiedemeier**: Formal analysis (equal); writing—review and editing (equal). **Marcel Müller**: Formal analysis (equal); writing—review and editing (equal). **Thomas Attin**: Conceptualization (equal), writing—review, and editing (equal). **Thomas Thurnheer**: Conceptualization (equal), writing—review, and editing (equal).

## Figures and Tables

**Figure A1:**
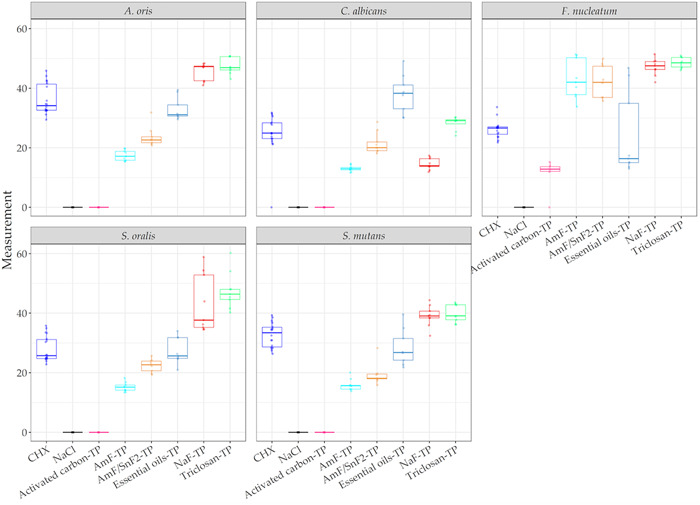
Boxplots with the median values and interquartile ranges of the disc diffusion assay present the antimicrobial efficacy of all six tested toothpaste suspensions

**Figure A2:**
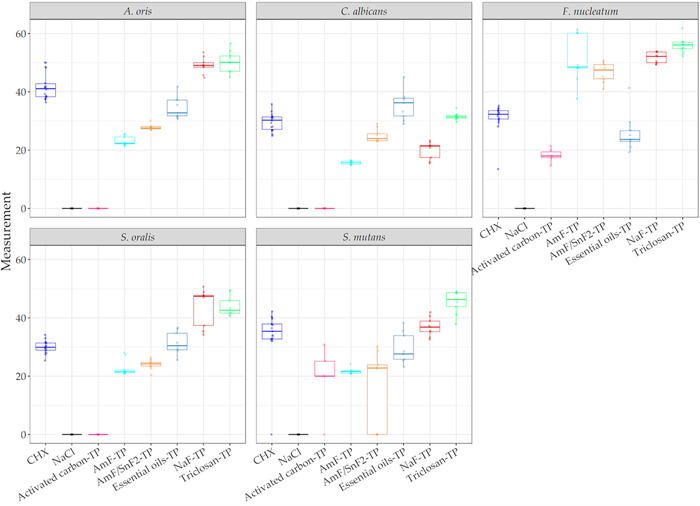
Boxplots with the median values and interquartile ranges of the plug diffusion assay present the antimicrobial efficacy of all six tested toothpaste suspensions

## Data Availability

All data generated or analyzed during this study are included in this published article except the supplemental table available at https://doi.org/10.6084/m9.figshare.19160843 – the raw data of the biofilm experiment (culture analysis), disc and plug diffusion assays, and the significant statistical differences of the biofilm experiment.

## APPENDIX A

Figures A1, A2, A3.
